# Supplementary material for: Adolescent HIV prevent and care framework: A global scoping review protocol- BSGH 006
Source: PLoS One. 2024 Aug 19;19(8):e0289994. doi: 10.1371/journal.pone.0289994 (PMC11332932; doi:10.1371/journal.pone.0289994)
Supplement: Attachment — Submitted filename: PONE-D-23-24131 Adolscent HIV.docx [file pone.0289994.s002.docx]

Adolescent HIV Prevent and Care Framework: A Global Scoping Review Protocol

Thank you for inviting me to review this protocol. The topic is very relevant and urgent in the fight against HIV.

The protocol is well written.

However, I had to read the protocol repeatedly trying to understand the methodology. While the reviewers propose to use the enhanced JBI methodology, the protocol lacks a review question, and related objectives. Rather, there a mention of an objective (p.g. 9 under outcome, and aim (p.g. 10 in the discussion), I suggest this should be included in the introduction.

I suggest in this protocol, the authors clearly list the steps they follow during the actual review. In addition, if necessary, limit the discussion to what they would include.

State the justification for inclusion of studies published in English only.
