# Supplementary material for: Adolescent HIV prevent and care framework: A global scoping review protocol- BSGH 006
Source: PLoS One. 2024 Aug 19;19(8):e0289994. doi: 10.1371/journal.pone.0289994 (PMC11332932; doi:10.1371/journal.pone.0289994)
Supplement: Attachment — Submitted filename: Response to Reviewers..doc [file pone.0289994.s003.doc]

Dear reviewers,

We have addressed the following changes mentioned by the reviewers in the table below

| **Reviewer Comment** | **Author Response** |
| --- | --- |
| Brief the article selection and initial screening. It appears that authors intend to perform initial screening just with Covidence (‘Duplicate removal and selection of articles that meet the inclusion criteria for the study will be performed using Covidence’). Covidence would be the platform to facilitate the process. | Covidence has been indicated as the platform through which the process of screening will be done. |
| Not clear as to what is exactly expected by mentioning ‘The extraction would include information on abstracts ….’. The data extraction needs to be from the full article content. | We have corrected this sentencing an indicate that data for the review will be gotten from the final screened full-texted. |
| The abstract ends abruptly. Please include briefings of data analysis and presentation, and discussion in the abstract. | We realized the abrupt nature the abstract was concluded and have included sections of the discussions and what we expect from the review process. |
| Strengthen the justification, late in the introduction, by highlighting the actual knowledge gap and it’s importance so emphasizing why this review needs to be conducted. | We have included the highlights of the knowledge gaps in the introductions section and importance of why these gaps need to be addressed. The first is the lack of comprehensive data that harmonizes research on HIV prevention and care among adolescents age 10-19, and the second, the lack of a global framework that captures the diverse dynamics in adolescent HIV prevention and care. |
| Present the review objective(s) / questions in a precise manner, as it appears to be much broader focusing on different aspects. Better to present the precise objectives separately. | We have presented in the manuscript the precise objectives of the study in the concluding parts of the introduction and discussion sections. |
| Indicate the adherence to JBI guidelines in conducting the review, apart from reporting according to PRISMA-ScR (both in abstract and main content). | We will adhere to the JBI guidelines in conducting the review. This has been indicated in the “**Process”** section under the “**Data extraction”** |
| Mention the inclusion and exclusion criteria separately so it is made clear. | We have separated the exclusion and inclusion criteria in sections “**Criteria for study inclusion” and “Criteria for study exclusion”** |
| Provide the search terms / search string that’ll be used in in the initial search. | The key search terms have been included in the section under electronic database searching. |
| Please indicate weather a quality appraisal of the articles will be performed or not, with reasoning if not. | we will not be doing an appraisal of the evidence as it “falls outside the realm of scoping review methodology” (Tricco et al., 2018) and does not align with the objectives of this scoping review.  Here are two papers that speak to appraisal in scoping reviews.     1. Tricco, A. C., Lillie, E., Zarin, W., O'Brien, K. K., Colquhoun, H., Levac, D., Moher, D., Peters, M. D. J., Horsley, T., Weeks, L., Hempel, S., Akl, E. A., Chang, C., McGowan, J., Stewart, L., Hartling, L., Aldcroft, A., Wilson, M. G., Garritty, C., . . . Straus, S. E. (2018). PRISMA Extension for Scoping Reviews (PRISMA-ScR): Checklist and Explanation. *Annals of Internal Medicine, 169*(7), 467-473. 10.7326/M18-0850      2. Peters, Micah D.J. BHSc, MA(Q), PhD1; Godfrey, Christina M. RN PhD2; Khalil, Hanan BPharm, MPharm, PhD3; McInerney, Patricia PhD4; Parker, Deborah5; Soares, Cassia Baldini RN, MPH, PhD6. Guidance for conducting systematic scoping reviews. International Journal of Evidence-Based Healthcare 13(3):p 141-146, September 2015. \| DOI: 10.1097/XEB.0000000000000050 |
| Avoid approximations within methodology and indicate what exactly would be done. E.g., ‘a search strategy from electronic search engines such as………….’ ‘The review will focus on related topics such as HIV transmission, safe…..’ ‘This review will not include certain types of publications such as’ | All approximations in the methods sections have been addressed in the manuscript. |
| Mention, in the analysis section, how the expected framework will be built using the data from the study. | We have indicated in the analysis section that “The proposed framework will be guided by data derived from the systematic review and recommendations from researchers in the diverse research fields in HIV prevention and care among adolescents aged 10-19 years. We will use data derived to serve as a checklist and a measure of the extent to which the framework considers the social differences, intersectionality, and political climates of adolescent globally” |
| Writing and typographic errors; Following are examples only and please check for all such typos and revise.  - Introduction 1st paragraph line 11 – Full stop - Introduction 4th paragraph – ‘The (38) has also proposed using psychosocial’ - World Health Organizations | We have addressed this review by correcting identified typographical errors, as well as those no mentioned by the reviewers. |
| Some places lack appropriate referencing; E.g., Although specific components, such as the psychosocial approach recommended by the World Health Organizations (2023), have been proposed for intervention, the context and individual characteristics play significant roles in determining how to approach HIV prevention and care among adolescents (REF). | We have included all citations where they need to be referenced. In the area where reviewer indicated |
| Some of the content is repetitive; (e.g., last paragraph of the discussion). Check and revise the manuscript to correct such repetitive information. | We have noted the repetitive nature of some of the sentencing in the manuscript and removed such information in the discussion and other sections of the paper. |
| Some content is redundant within sentences. E.g., ‘To ensure up-to-date information, only studies published after 2013 will be included, and earlier ones will be excluded.’ Here, the ‘earlier ones will be excluded’ appear redundant. | We have removed all redundant wording in the manuscript. In the example given by the reviewer “and earlier ones will be excluded’ was deleted. |
| **Reviewer 2** |  |
| The topic is very relevant and urgent in the fight against HIV.The protocol is well written. However, I had to read the protocol repeatedly trying to understand the methodology. While the reviewers propose to use the enhanced JBI methodology, the protocol lacks a review question, and related objectives. Rather, there a mention of an objective (p.g. 9 under outcome, and aim (p.g. 10 in the discussion), I suggest this should be included in the introduction. | We have included the review questions/gaps and the proposed objectives of the study in the last paragraph of the introduction. |
|  |  |

Sincerely

Osman Wumpini Shamrock PhD, MPhil

School of Nursing, University of Rochester

225 Crittenden Blvd, Rochester, NY, 14642

+1 (607) 761 8189
