# Supplementary material for: Adolescent HIV prevent and care framework: A global scoping review protocol- BSGH 006
Source: PLoS One. 2024 Aug 19;19(8):e0289994. doi: 10.1371/journal.pone.0289994 (PMC11332932; doi:10.1371/journal.pone.0289994)
Supplement: Attachment — Submitted filename: reviewers reponse letter.docx [file pone.0289994.s004.docx]

Dear reviewers,

We have addressed the following changes mentioned by the reviewers in the table below:

| Reviewer comments | Response |
| --- | --- |
| Reviewer #1: The Authors have addressed the previous comments / suggestions mostly. However, the minor comment 'Avoid approximations within methodology and indicate what exactly would be done' has not been properly addressed, despite being indicated as addressed in the response letter. | We have gone through the methodology section and removed all approximations, indicating exactly what will be done in the study. |
| Reviewer #3: The rationale does not come out clearly. Authors need to succinctly explain why this undertaking is necessary. | We have highlighted adolescent reproductive and sexual health as a significant public health concern, and considering the cumulative facts presented in the introduction of this protocol pertaining to our population and targeted age group (10-19 years), we believe that we have sufficiently laid the groundwork for the rationale. With this build-up, we are confident in presenting the rationale for undertaking efforts to address HIV prevention and care among adolescents, along with our proposal. |
| Some of the statements need to be backed up with references. An example is stating that 'Among adolescents, HIV/AIDs remain the second leading cause of death globally'. Please check that this is correct, and the citation provided does not help much. | We accept the reviewer’s recommendation and rephrased the statement to read “Among adolescents, HIV/AIDs remain a significant cause of death globally”. We have also updated the references to back this claim. (Judd et al. 2018; Amour et al. 2022; Jerene et al. 2019; UNICEF. 2021) |
| A few texts are difficult to interpret and, in some cases, come off as overstated. Some examples:  ''Sex stigma in some communities deter adolescents from accessing HIV care - A bit difficult to comprehend. | We accept this recommendation and rewritten this sentence different, “Stigma related to HIV and sexuality in certain communities may discourage adolescents from seeking HIV care.” |
| Determining appropriate antiretroviral (ART) dosages for sustained suppression in adolescents is challenging - Does not come off as factual. | We have clarified what we meant by the statement by indicating that growth spurt common among adolescents may be a factor that could affect the right amount of ART dose administered. The appropriate reference has been included. |
| The World Health Organizations (2023) has also proposed using psychosocial techniques to support preventive measures for HIV among adolescents - any citation?” | Th e We have citation in the text and reference section;  “ World Health Organization. Integrating psychosocial interventions and support into HIV services for adolescents and young adults. Published online June 14, 2023. |
| Authors need to check the feasibility of outcomes. For instance, is it feasible to generate a comprehensive data analysis that would consider such diverse social factors such as geography, race, sex, age, gender, education, religion, sexual orientation, occupation, nationality, marital status, socio-economic status, disability, and immigration status? | We greatly acknowledge the reviewer’s comment but stick to the proposal as indicated in the protocol. |
| Finally, the authors speak to the dissemination of findings but not the ready availability of results and comprehensive data. Is there an existing repository that can house such data? | The University of Rochester Research Repository (URRR) offers a place to deposit research results and comprehensive data.. |

Sincerely

Osman Wumpini Shamrock PhD, MPhil

School of Nursing, University of Rochester

225 Crittenden Blvd, Rochester, NY, 14642

+1 (607) 761 8189
